# Downregulation of lncRNA SLC7A11-AS1 decreased the NRF2/SLC7A11 expression and inhibited the progression of colorectal cancer cells

**DOI:** 10.7717/peerj.15216

**Published:** 2023-04-14

**Authors:** Tian Wang, Si Liang, Yajing Li, Xiyu Wang, Hongjie Wang, Jiguang Guo, Ming Li

**Affiliations:** 1 Department of Anesthesiology, Affiliated Hospital of Hebei University, Baoding, Hebei, China; 2 College of Clinical Medicine, Hebei University, Baoding, Hebei, China; 3 Endoscopic Diagnosis and Treatment Center, Affiliated Hospital of Hebei University, Baoding, Hebei, China; 4 School of Basic Medical Sciences, Hebei University, Baoding, Hebei, China

**Keywords:** Colorectal cancer, SLC7A11-AS1, NRF2, SLC7A11, Reactive oxygen species

## Abstract

Colorectal cancer (CRC) is ranked as the second leading cause of cancer-related death worldwide. Many abnormally expressed long non-coding RNAs (lncRNAs) in CRC were identified with the development of next-generation sequencing, most functions of which are largely unclear. In this study, we report that the lncRNA SLC7A11-AS1 was significantly overexpressed in CRC by analyzing TCGA database and 6 pairs of clinical samples. High SLC7A11-AS1 level was related to poor CRC overall survival and SLC7A11-AS1 knockdown could inhibit the proliferation, migration and invasion of CRC cell lines. Furthermore, we found there was a positive correlation between the expression of SLC7A11-AS1 and its’ sense transcript SLC7A11. In HCT-8 cells, SLC7A11-AS1 knockdown decreased expression of both SLC7A11 and the nuclear level of NRF2, which happens to be the activator of SLC7A11 transcription. Interestingly, in SLC7A11-AS1 overexpressed CRC tissues, SLC7A11 and NRF2 were also upregulated. Moreover, the ROS levels increased with SLC7A11-AS1 knockdown in HCT-8 cells. And the down regulated expression of SLC7A11 and lower ROS level causing by SLC7A11-AS1 knocked down could be relieved by overexpressed NRF2. These results suggested that upregulated SLC7A11-AS1 might promote the formation and progression of CRC by increasing the expression of NRF2 and SLC7A11, which decreases the ROS level in cancer cells. Therefore, SLC7A11-AS1 could be a potential therapeutic target and diagnostic marker of CRC.

## Introduction

As estimated by the GLOBOCAN, there were 1.9 million newly diagnosed colorectal cancer (CRC) cases and 935 thousand CRC-related deaths in 2020 (Sung et al., 2021). CRC was ranked as the second leading cause of malignant tumor deaths and the third most common malignancy. Although the incidence of CRC among adults aged more than 50 declined in some countries due to the implementation of colonoscopy screening, a 1%–4% incidence increase was observed in the population aged less than 50 (Patel et al., 2022). Overall, the incidence rate of CRC was rising with the development of society (Fidler, Soerjomataram & Bray, 2016). Surgical resection combined with chemotherapy is still the first choice for patients, but approximately 30% of patients will have a recurrence (van der Stok et al., 2017). Therefore, there is an urgent need for a better understanding of CRC pathogenesis to provide new therapeutic targets and diagnostic markers.

Long noncoding RNAs (lncRNAs) are noncoding RNA molecules larger than 200 nucleotides. They are important regulators of cancer formation and development. Recent studies indicated that the dysfunction of lncRNA SLC7A11-AS1 is associated with various types of cancer. The upregulated SLC7A11-AS1 could block the ubiquitination and degradation of the nuclear factor E2-related factor 2 (NRF2) by interacting with *β*-transducin repeat containing protein 1 (*β*-TRCP1), which would, in turn, decrease the reactive oxygen species (ROS) levels and mediate the drug resistance of pancreatic ductal adenocarcinoma (Yang et al., 2020). Liu et al. (2020) reported that SLC7A11-AS1 was also increased in lung cancer and associated with cancer progression. Knockdown of SLC7A11-AS1 suppressed the proliferation and invasion of lung cancer cells by sponging miR-4775 to enhance the expression of TRAF interacting protein (TRAIP) (Liu et al., 2020). SLC7A11-AS1 was also elevated in hepatocellular carcinoma and likely participated in metastasis (Liang et al., 2020). Contrary to previous studies, in gastric cancer and epithelial ovarian cancer, SLC7A11-AS1 decreased (Yuan, Liu & Song, 2017; Luo et al., 2022). Although various lncRNAs have been reported as the diagnostic or prognostic markers for CRC (Kishore & Karunagaran, 2022), the role of SLC7A11-AS1 in CRC has not been elucidated.

LncRNAs positively regulated the expression of its sense transcripts have been observed in glioma and breast cancer (Wang et al., 2021; Zheng et al., 2021). SLC7A11 is the adjacent sense transcript of SLC7A11-AS1, which is a key subunit of glutamate/cysteine antiporter system Xc ^−^ to synthesize the main intracellular antioxidant glutathione (Zuo et al., 2022). It has been reported that the expression of SLC7A11 was upregulated by NRF2 to counteract the increased oxidative stress in cancer cells (Habib et al., 2015; Shin et al., 2017). Feng et al. (2021) reported that high expression of SLC7A11 and nuclear level of NRF2 not only positively correlated with each other in esophageal squamous cell carcinoma (ESCC) tissues, but also associated with lymph node metastasis and lower overall survival. Blocking the activity of NRF2 by different kinds of natural products will compromise SLC7A11 expression and suppress the progression of head and neck cancer and glioma (Yu et al., 2020; Cai et al., 2021). In addition, Yang et al. (2020) proved that SLC7A11-AS1 decreased the ROS level by stabilizing NRF2 in pancreatic cancer. However, SLC7A11-AS1 was suggested to negatively regulate the expression of SLC7A11 in the research of gastric cancer and epithelial ovarian cancer (Yuan, Liu & Song, 2017; Luo et al., 2022). Therefore, we aimed to explore the relationship between SLC7A11-AS1 and its sense transcript SLC7A11 in CRC, which has not been investigated.

In the present study, we identified that SLC7A11-AS1 was significantly overexpressed in colon adenocarcinoma (COAD) tissues and related to the poor overall survival of the patients. Silencing the expression of SLC7A11-AS1 will suppress the proliferation and migration of CRC cells and elevate the intracellular ROS level. Moreover, we found that SLC7A11-AS1 might positively regulate the expression of SLC7A11 by increasing the protein level of NRF2 in CRC.

## Materials & Methods

### Patients

A total of six pairs of fresh CRC tissues and normal adjacent tissues were collected from the Affiliated Hospital of Hebei University in 2021 and stored at −80 °C. All the patients who participated in the study had written informed consents, got a pathological diagnosis, and had no treatment before surgery. Three men and three women were involved in the study with an age range of 56–80 years and a mean age of 70 years.

### Bioinformatics analysis

The pan-cancer analysis of the expression of SLC7A11-AS1 was performed by the ncFANs v2.0 website (http://ncfans.gene.ac/) (Zhang et al., 2021). The transcriptome data of COAD patients has been got accessed from The Cancer Genome Atlas (TCGA) dataset (https://portal.gdc.cancer.gov/) for further analysis. The prognostic performance of SLC7A11-AS1 in COAD was assessed by Gene Expression Profiling Interactive Analysis (GEPIA) website (Tang et al., 2017). The expression of SLC7A11-AS1 in the normal and COAD tissues was compared by the ggbetweenstats package with Welch’s *t*-test in R software (version 4.1.0). The Pearson correlation coefficient between the expression of SLC7A11-AS1 and the other genes was also calculated by the cor.test function of the R software and visualized by the Cytoscape software (version 3.7.2). R <−0.5 or *R* > 0.5 and *p* < 0.05 were considered to be relevant. The expression of genes that related to SLC7A11-AS1 in COAD patients was visualized by using the pheatmap package in R software. The mutational profiles of the related genes in COAD patients were identified by the maftool package in R software to create the mutation annotation format from TCGA COAD database (Mayakonda et al., 2018). Moreover, the Gene Ontology (GO) functional enrichment analysis of the related genes was carried out by the Metascape database (https://metascape.org/gp/index.html#/main/step1) (Zhou et al., 2019).

### Cell culture and transfection

The human CRC cell line HCT-8 was donated by Dr. Nan Chen the associate professor of the School of Basic Medical Sciences of Hebei University. The normal colon epithelial cell FHC and CRC cell HCT-116 were provided by Dr. Yan Qin the associate professor of Affiliated Hospital of Hebei University. All the cells were cultured in RPMI-1640 medium (Wisent Biotechnology, Nanjing, China) with 10% FBS (Biological Industries, Beit Haemek, Israel), containing 100 U/mL penicillin and 100 mg/mL streptomycin (Solarbio, Beijing, China). All cells were incubated at 37 °C in a humidified atmosphere of 5% carbon dioxide. GenePharma (Shanghai, China) synthesized antisense oligonucleotides (ASO), the NRF2 overexpression vector was purchased from Obio Technology (Shanghai), both of which were transiently transfected into cells with Lipofectamine 3000 reagent (Invitrogen, CA, USA). as directed by the manufacturer. The sequences of the SLC7A11-AS1 ASO and negative control (NC) ASO were as follows: SLC7A11-AS1 ASO 5′- UGACUAGGTAGGAAGGGUGC −3′; NC ASO 5′- GCGUATTATAGCCGATTAAC -3′.

### Wound healing assay

When the HCT-8 cells arrived at approximately 90% confluence, ASOs were transiently transfected into the cultured cells in 6-well plates (Jet Biofil, Guangzhou, China). 200-µL pipette tip was used to scrape the cell monolayer to create a wound gap after transfection for 24 h. The wounded monolayers were then cultured with RPMI-1640 medium containing 1% FBS and observed at 24 h and 48 h. The scratches were captured by microscope using FLUOCA FCSnap software (version 1.1.19627) and five fields were randomly selected for each scratch wound. The gap lengths were measured with Image J software (version Java 1.8.0_172).

### Transwell migration and invasion

After the transfection of ASOs into CRC cells for 24 h, 1 × 10^4^ cells per well were seeded in the upper chamber of the tissue culture plate insert (Jet Biofil, Guangzhou, China) in 100 µl of serum-free medium. At the same time, 600 µl of complete medium was added to the lower chamber of the tissue culture plate insert. After being incubated for 24 h at 37 °C, cotton swabs were used to remove the cells remaining at the upper surface of the membrane, and the migrated cells on the lower surface of the membrane were fixed. 0.1% crystal violet solution was used to stain the migrated cells after fixing them with 4% paraformaldehyde. 4 × 10^4^ cells per well were seeded in the upper chamber coated by Matrigel (BD Biosciences) and incubated for 48 h, when doing the invasion assay with the same procedure. The cells that passed through the membrane were observed by microscope and captured by FLUOCA FCSnap software (version 1.1.19627).

### Cell counting kit-8 assay

Cell viability was detected by Cell Counting Kit-8 (CCK8, Dojindo, Japan) assay in accordance with the manufacturer’s protocols. After the transfection of ASOs into CRC cells for 24 h, CRC cells were seeded at a density of 5 × 10^3^ perwell with 100 µl complete medium in 96-well plates (Jet Biofil, Guangzhou, China). After cultured for 24, 48, and 72 h, CCK-8 reagent with a volume of 10 µl was added to each well and then incubated for 2 h. The absorbance was measured at 450 nm using the microplate reader (Bio-Rad, CA, USA). The ability of cell proliferation was represented by the optical density (OD).

### Colony formation assay

After the transfection of ASOs for 24 h, CRC cells were inoculated into the 6-well plates at a density of 1 × 10^3^ cells per well in a complete medium and cultured for 15 days to allow the formation of colonies. Afterwards, the plates were washed in cold PBS and fixed at room temperature in 4% polyformaldehyde. 0.1% crystal violet solution was used to stain the colonies for 30 min at room temperature. The colonies were counted by Image J software (version Java 1.8.0_172). Colony formation rate was calculated by the number of each SLC7A11-AS1 ASO/the number of NC ASO ×100%.

### Western blot

Total proteins were extracted from tissues or HCT-8 cells with RIPA buffer (Biomed, Beijing, China) containing one mmol/L PMSF (Solarbio, Beijing, China). The protein concentration was detected using a BCA protein assay reagent kit (Biomed, Beijing, China). Separations of proteins were carried out using 12% polyacrylamide gel electrophoresis, and then the proteins were transferred to nitrocellulose filter membranes. After blocking in 5% skim milk for 1 h, the anti-NRF2 antibody (1:1000, GeneTex, CA, USA), anti-SLC7A11 antibody (1:1000; Zen-Bioscience, Chengdu, China), and anti- *β* actin antibody (1:3000; Proteintech, Wuhan, China) were used as the primary antibodies in this study for overnight incubation at 4 °C. Afterwards, the membranes were incubated at room temperature for 1 h with the corresponding secondary antibodies. Image J software (version Java 1.8.0_172) was used to analyze the protein bands after they were visualized using enhanced chemiluminescence (ECL; Biomed, Beijing, China).

### Extraction of RNA and quantitative reverse transcription-PCR (RT-qPCR)

Total RNA was extracted from HCT-8 cells or tissues using TRNzol Universal reagent (TIANGEN, Beijing, China). cDNA was synthesized using the PrimeScript II 1st Strand cDNA Synthesis Kit (Takara, Shiga, Japan). RT-qPCR was subsequently conducted by using UltraSYBR One Step RT-qPCR Kit (CWBIO, Jiangsu, China) on Roche LightCyler 96. Normalization of each gene was done against 18S ribosomal RNA (18S) or GAPDH at transcript levels. The comparative cycle threshold (Ct) method (2^−ΔΔCt^) was applied to calculate relative expression levels of genes. The sequence of the primers used in the RT-qPCR was synthesized by Sangon (Shanghai, China) and shown in Table 1.

### Reactive oxygen species quantitation and visualization

HCT-8 cells were detached by trypsin (Solarbio, Beijing, China), seeded in a 96-well culture plate (Wohong Biotechnology, Shanghai, China) and cultured for 24 h, which has been transfected with ASOs for 24 h. Dihydroethidium, DHE (Beyotime, Shanghai, China) was diluted 1:1000 in serum-free medium to the final concentration of 10 µM and used to replace the culture medium in a 96-well culture plate to detect the ROS level. Using a fluorescence microplate reader (Thermo Fisher, Waltham, MA, USA), the fluorescence intensity was examined at wavelengths of 485 nm for excitation and 590 nm for emission.

After being transfected with ASOs for 24 h, HCT-8 cells were collected and seeded in 24-well plates (Jet Biofil, Guangzhou, China). After 24 h, the cell culture medium was removed and replaced by 500 µl diluted DHE for 20 min incubation. Cells were then visualized by fluorescence microscopy (Leica DMi8, Wetzlar, Germany) with Leica Application Suite X software (version 3.1.1.15751).

**Table 1:** The sequence of the Primers used in the study.

Primers for RT-qPCR	Sequence (5′–3′)
SLC7A11-AS1	Forward: AGCCTGGGTGATAAAGTG Reverse: TAAGCCCTCAATGGATAG
GAPDH	Forward: TGCACCACCAACTGCTTAGC Reverse: GGCATGGACTGTGGTCATGAG
18S	Forward: AACTTTCGATGGTAGTCGCCG Reverse: CCTTGGATGTGGTAGCCGTTT
MT-RNR1	Forward: CCTCCCCAATAAAGCTAAAA Reverse: GCTATTGTGTGTTCAGATAT
U99	Forward: CCTCCTTTTCTTGGCGGGGA Reverse: CGTTTGAGGATAGAACCAGC
SLC7A11	Forward: TGTGTGGGGTCCTGTCACTA
	Reverse: CAGTAGCTGCAGGGCGTATT

### Cytoplasmic and nuclear RNA and protein extraction

Separation of cytoplasmic and nuclear RNA and protein were performed by using the Nuclear and Cytoplasmic Protein Extraction Kit (Beyotime, Shanghai, China) plus TRNzol Universal reagent or RIPA buffer. HCT-8 cells were harvested with trypsin-EDTA and centrifugation at 500 g for 3 min. The supernatant was removed and discarded as much as possible. 200 µl cytoplasmic extraction reagent A (for 2 × 10^6^ cells) (with RNase inhibitors for RNA isolation) was used to resuspend the precipitate. After incubation for 20 min on ice, 10 µl cytoplasmic extraction reagent B was added to the tube and centrifuged at 12,000 g for 10 min, 4 °C. The supernatant was moved to a new tube and the TRNzol Universal reagent was used to extract the cytoplasmic RNA or RIPA buffer for protein extraction. The precipitate was also exacted with TRNzol Universal reagent to harvest the nuclear RNA or RIPA buffer for protein extraction. We assessed the quality and quantity of the RNA using the NanoDrop (Thermo Fisher, Waltham, MA, USA) at 260 and 280 nm wavelengths.

### Statistical analysis

A minimum of three independent experiments were conducted for each experiment. The student’s *t*-test was performed using GraphPad Prism Version 7 for the analysis between two groups (San Diego, CA, USA). Analysis of variance (ANOVA) was used for the comparison of more than two groups. The data were expressed as the mean ±SEM. *p* < 0.05 was considered statistically significant.

## Results

### SLC7A11-AS1 was highly expressed in CRC and associated with a poor prognosis

**Figure 1:**
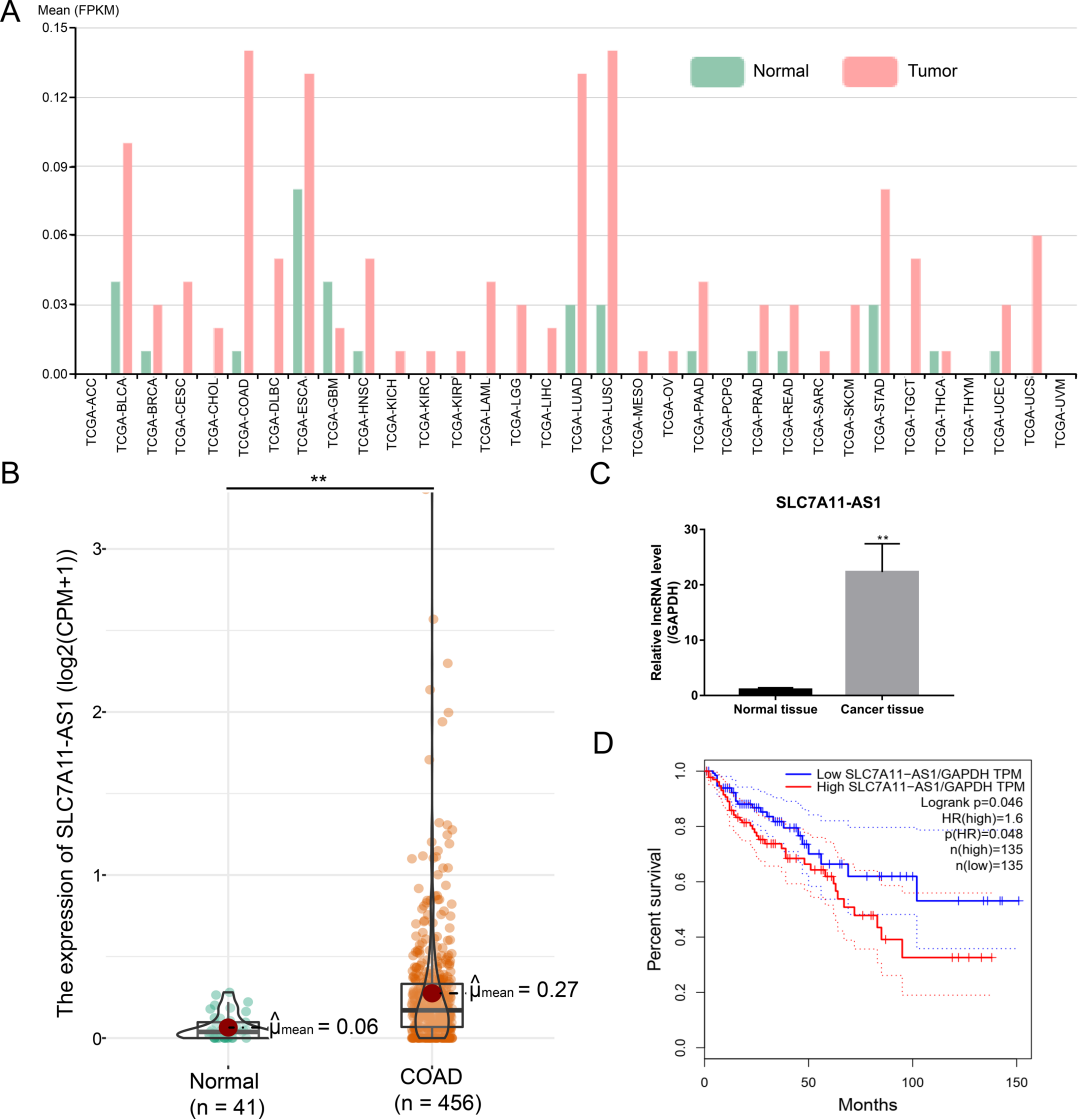
Over expression of SLC7A11-AS1 was detected in various kinds of cancer and related to the unfavorable overall survival of CRC. (A) The expression pattern of SLC7A11-AS1 in 33 different kinds of cancers by analyzing the TCGA database. The full names of TCGA cancer name abbreviations was provided in Table S2. (B) The expression of SLC7A11-AS1 in 456 TCGA COAD tissues compared between the 41 normal tissues. (C) The level of SLC7A11-AS1 in our CRC tissues compared with the normal adjacent tissues. (D) Elevated SLC7A11-AS1 level was associated with dismal prognosis in COAD patients by analyzing the GEPIA website. FPKM, fragments per kilobase of exon model per million mapped fragments; CPM, counts of exon model per million mapped reads; TPM, transcripts per kilobase of exon model per million mapped reads; ^∗∗^*p* < 0.01.

After analysis by the ncFANS (version 2.0) website and the TCGA database, we found that the expression of SLC7A11-AS1 was significantly elevated in various types of cancers including CRC (Figs. 1A and 1B). SLC7A11-AS1 was also significantly overexpressed in our CRC tissues compared to normal tissues (Fig. 1C and Table S1). Moreover, high SLC7A11-AS1 level was related to lower overall survival (Fig. 1D).

### Silencing the SLC7A11-AS1 expression suppressed the proliferation of CRC cells

**Figure 2:**
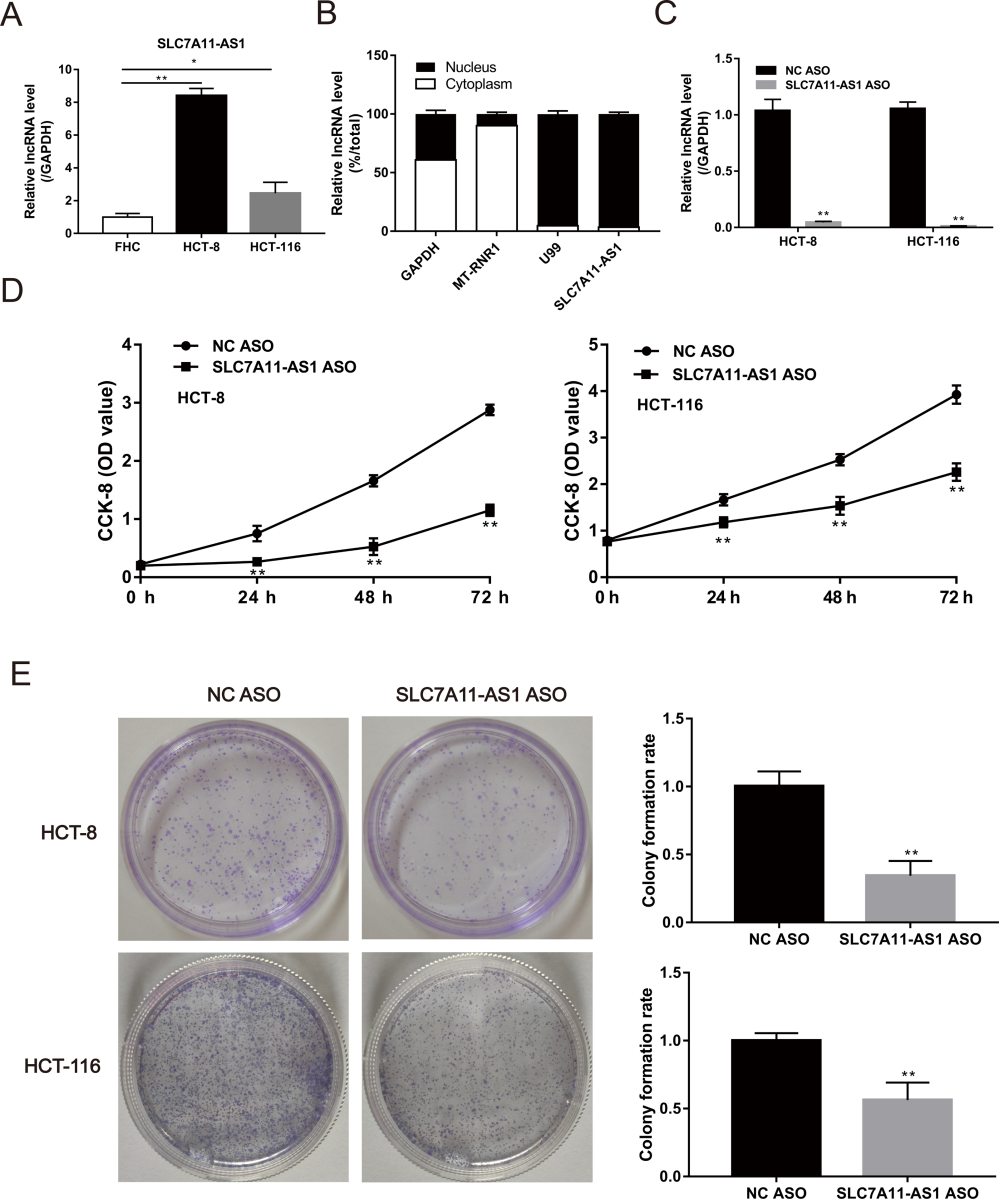
The proliferation ability of CRC cells was weakened by SLC7A11-AS1 knockdown. (A) The expression of SLC7A11-AS1 was significantly higher in the CRC cells than in the normal FHC cells. (B) The distribution of SLC7A11-AS1 in the cytoplasm and nuclear. U99 was used as the nuclear marker and MT-RUR1 as the cytoplasmic marker. The mRNA of GAPDH was detected both in the cytoplasm and nuclear. (C) The expression of SLC7A11-AS1 was knocked down by SLC7A11-AS1 ASO. (D) The viability of CRC cells was determined by CCK-8 assay after silencing the expression of SLC7A11-AS1. (E) The representative graphics of colony formation were displayed. The average colony formation rate after transfected SLC7A11-AS1 ASO was compared with NC ASO. ^∗^*p* < 0.05, ^∗∗^*p* < 0.01.

The expression of SLC7A11-AS1 in HCT-8 and HCT-116 cells was significantly higher than the normal FHC cell (Fig. 2A and Table S1). Then, we confirmed that SLC7A11-AS1 was mainly distributed in the cell nucleus by separating the cytoplasmic and nuclear total RNA in HCT-8 cell (Fig. 2B and Table S1). Therefore, we knocked down the expression of SLC7A11-AS1 by transfecting the SLC7A11-AS1 ASO into CRC cells (Fig. 2C and Table S1). We found that silencing SLC7A11-AS1 expression significantly attenuated the HCT-8 and HCT-116 cell proliferation measured by the CCK-8 assay and the colony formation experiment (Figs. 2D, 2E and Table S3).

### SLC7A11-AS1 knockdown inhibited the migration of CRC cells

**Figure 3:**
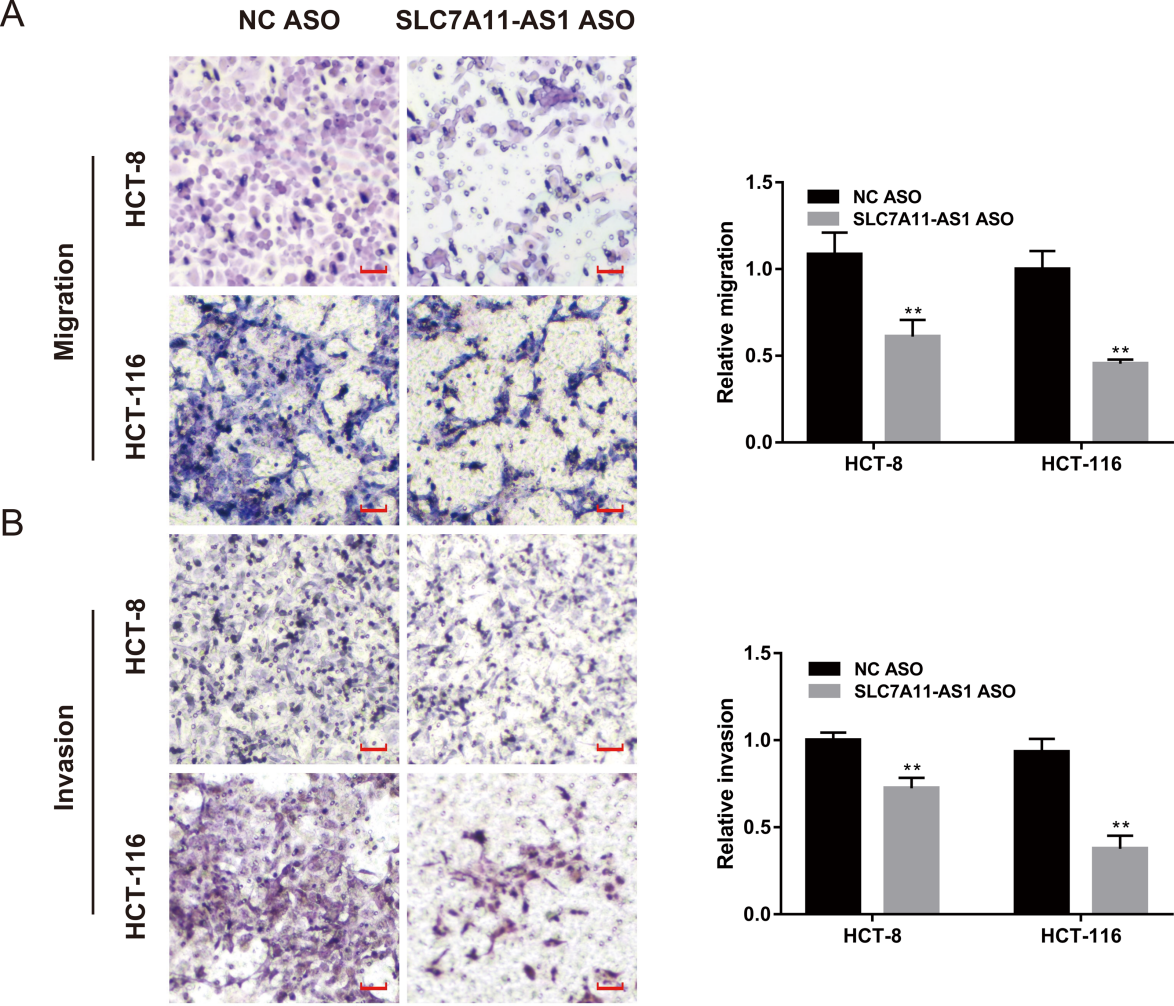
The migration and invasion of CRC cells decreased with SLC7A11-AS1 knockdown. (A, B) The migration assay and invasion assay were carried out to assess the effect of silencing SLC7A11-AS1 on CRC cells. ^∗^*p* < 0.05, ^∗∗^*p* < 0.01. The scale bar is 50 µm.

We also evaluated the effect of SLC7A11-AS1 knockdown on cell migration by performing the wound healing assay. We found that the migration of HCT-8 cells was suppressed by low expression of SLC7A11-AS1 at 24 h and 48 h after SLC7A11-AS1 ASO transfection (Fig. S1 and Table S3). We further confirmed our results by transwell assay, which exhibited that silencing SLC7A11-AS1 expression hindered the ability of HCT-8 and the HCT-116 cells migration (Fig. 3A and Table S3). A similar inhibitory effect was also observed in the invasion array of HCT-8 and the HCT-116 cells after knocking down the expression of SLC7A11-AS1 (Fig. 3B and Table S3).

### The SLC7A11-AS1 expression positively correlated with the expression of SLC7A11 in CRC

**Figure 4:**
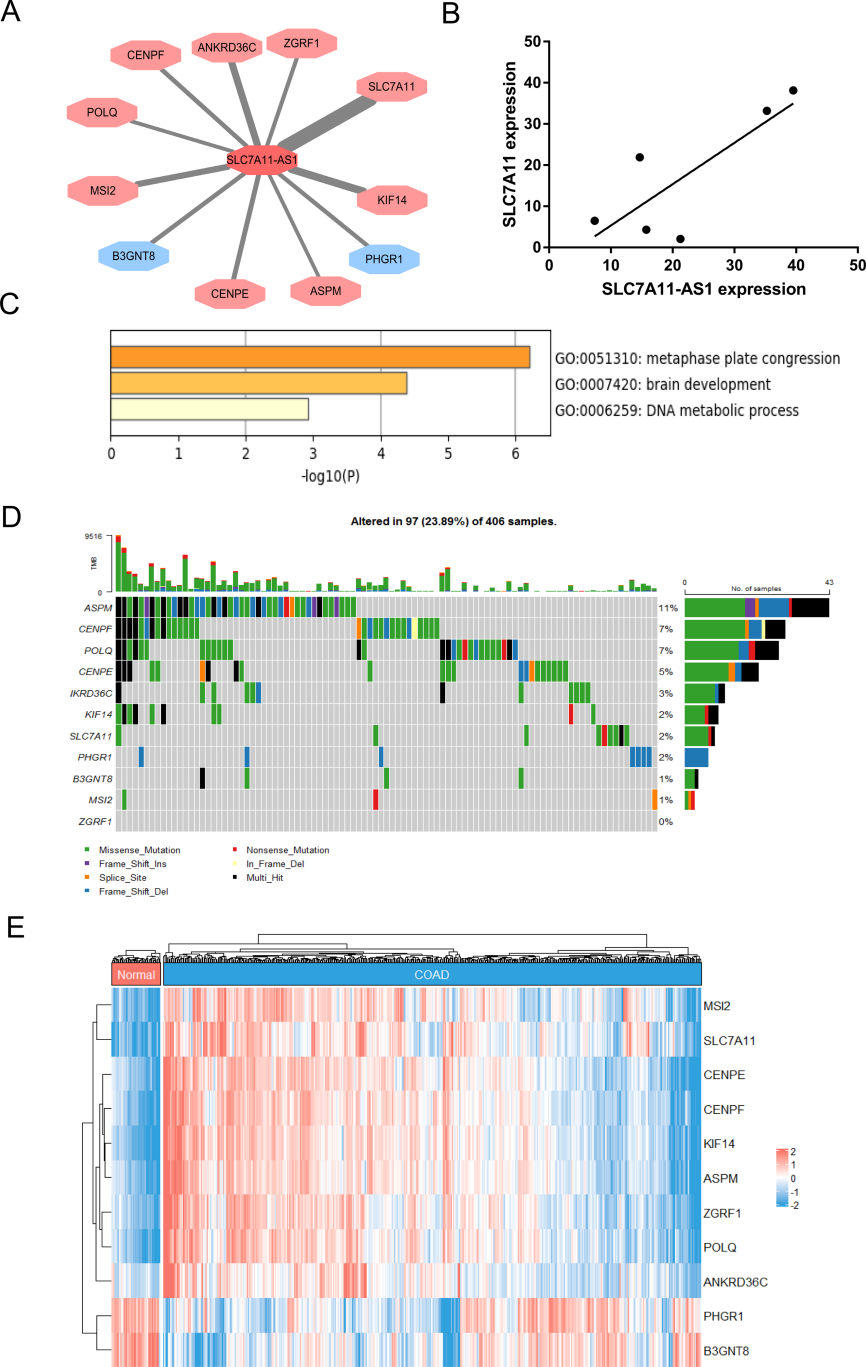
Identification of the genes related to SLC7A11-AS1 by bioinformatics analysis. (A) The network showed that the expression of the genes was associated with the expression of SLC7A11-AS1. The genes in the red octagon had a positive correlation with SLC7A11-AS1, while the blue one had a negative correlation. The thinner the line was, the higher the |R|  was. (B) The mRNA expression of SLC7A11 was positively associated with SLC7A11-AS1 in our clinical tissues. *R* = 0.8059; *p* = 0.0529. (C) The representative GO terms enriched by the SLC7A11-AS1 related genes. (D) Genetic alteration analysis of the related genes. (E) The heat map illustrating the expression pattern of the related genes.

To identify the target genes of SLC7A11-AS1 in CRC, we performed the correlation analysis using the TCGA COAD transcriptome dataset. The results showed that the expression of SLC7A11-AS1 was positively related to SLC7A11, POLQ, ASPM, CENPF, KIF14, ZGRF1, CENPE, MSI2, ANKRD36C, and negatively associated with B3GNT8, PHGR1 (Fig. 4A). We also detected the expression relationship between SLC7A11-AS1 and the mRNA level of SLC7A11 in our clinical tissues. The positive relation was confirmed in our clinical tissues, as shown in Fig. 4B (*R* = 0.8059). Enrichment analysis indicated that these genes were mostly enriched in the GO terms of metaphase plate congression (containing CENPE, CENPF, KIF14, ASPM, and MSI2) and brain development (including CENPF, KIF14, SLC7A11, ASPM) (Fig. 4C). The tumor mutation burden (TMB) of these genes was also analyzed, and ASPM, CENPF, and POLQ had higher mutation rates than the other genes (Fig. 4D). The expression profile of these related genes was shown by the heatmap in Fig. 4E, and we found that SLC7A11 was significantly upregulated in the TCGA COAD dataset in accordance with the expression profile of SLC7A11-AS1 (Fig. 4E).

### NRF2 over expression could rescue the SLC7A11 expression and ROS level after SLC7A11-AS1 knocked down

**Figure 5:**
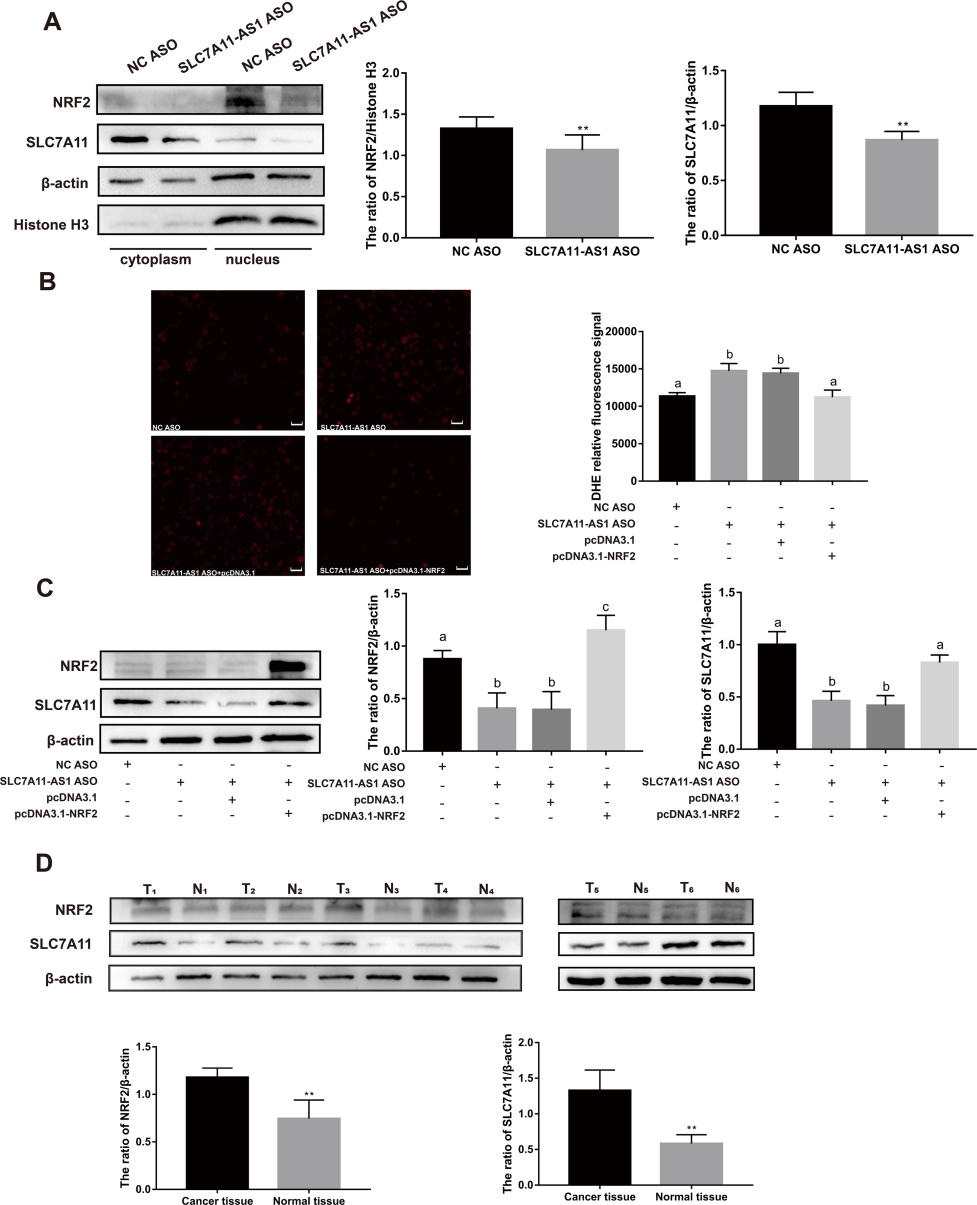
SLC7A11-AS1 regulated the ROS level by NRF2/SLC7A11 signaling pathway. (A) Knocked down the expression of SLC7A11-AS1 led to decreased cytoplasmic protein level of SLC7A11 and nuclear level of NRF2 in HCT-8 cells. (B) The representative fluorescence images and quantitive analysis of the DHE assay showing that the increased ROS level was restored by NRF2 overexpression after silencing SLC7A11-AS1 expression. (C) Downregulated SLC7A11 expression was relieved by NRF2 overexpression after SLC7A11-AS1 knocked down. (D) NRF2 and SLC7A11 were over expressed in the clinical CRC tissues compared with the normal tissues. N, normal tissue; T, cancer tissue. ^∗^*p* < 0.05, ^∗∗^*p* < 0.01. The significant difference was observed between the groups with difference letters, and no significant difference between the groups of the same letter. The scale bar is 50 µm.

To further investigate the relationship between SLC7A11-AS1 and SLC7A11, we silenced the lncRNA SLC7A11-AS1 by its’ ASO. We found that SLC7A11-AS1 knockdown decreased the expression of SLC7A11 in HCT-8 cells (Fig. 5A and Fig. S2). Since SLC7A11-AS1 could stabilize NRF2 in pancreatic cancer (Yang et al., 2020) and SLC7A11 is also a common target of NRF2 (Fan et al., 2017), we further analyzed the effect of SLC7A11-AS1 on the NRF2 expression. We found silencing SLC7A11-AS1 could decrease the nuclear level of NRF2 and increase the ROS level (Figs. 5A, 5B and Table S3). However, this effect could be relieved by NRF2 overexpression (Figs. 5B, 5C and Table S3). Meanwhile, we found that there were upregulated SLC7A11 and NRF2 expression levels in the clinical CRC tissues with overexpressed SLC7A11-AS1 (Fig. 5D). Therefore, a high SLC7A11-AS1 level might help to clear the extra ROS by upregulation of the NRF2/SLC7A11 antioxidant system.

## Discussion

Until now, there are still no non-invasive biomarkers for the diagnosis of CRC. More and more research indicated that lncRNAs might be a potent non-invasive biomarker for CRC scanning (Yang et al., 2021). Xu et al. (2020) identified that plasma lncRNA SNHG1 could be a CRC marker for diagnosis. We noticed that some lncRNAs, such as CCAT1, PVT-1, MEG3, and H19 that proved to be significantly upregulated in CRC tissues compared with the normal tissues, were found to be promising plasma diagnostic biomarkers, especially for distinguishing early-stage CRC (Liu et al., 2019). Here, we identified that the expression of lncRNA SLC7A11-AS1 had significantly elevated in the CRC tissues than in the normal colon tissues for the first. Moreover, a high SLC7A11-AS1 level is associated with poor CRC patients’ survival and might lead to increased NRF2 and SLC7A11 expression to keep the redox homeostasis.

In order to elucidate the function of SLC7A11-AS1 in CRC, we silenced the expression of SLC7A11-AS1, which led to the inhibition of CRC cell proliferation, migration and invasion and elicited decreased NRF2 levels and high ROS levels. Similarly, Yang and his team have established that SLC7A11-AS1 knockdown would promote NRF2 degradation, ultimately leading to an increase in ROS levels and the sensitivity to gemcitabine in pancreatic cancer (Yang et al., 2020). The role of ROS in the formation and development of cancers, including CRC, is a double-edged sword. The proliferation, angiogenesis, and epithelial-to-mesenchymal transition (EMT) of cancer cells are fostered by a low or moderate level of ROS. High level of ROS can have an antitumor effect which damages cellular proteins, DNA, and lipids in the opposite (Sorolla et al., 2021). A majority of chemotherapeutic drugs greatly elevated the ROS levels of cancer cells so as to disrupt the redox homeostasis (Yang et al., 2018). In order to coordinate with the proliferation and metastasis of cancer cells, the metabolic rate is greatly elevated leading to the genesis of ROS increased (Perillo et al., 2020). Therefore, the antioxidant system is upregulated to clear the extra ROS and keep the redox homeostasis (Fan et al., 2017; Li et al., 2019; Yuan et al., 2021). NRF2 is a pivotal transcription factor in the modulation of antioxidant defense and redox homeostasis (Rojo de la Vega, Chapman & Zhang, 2018). The target genes of NRF2 mainly are cytoprotective genes and contains the antioxidant response element for its binding and activation (Nakamura & Takada, 2021). Over expression of NRF2 has been observed in various type of cancers containing CRC and promotes the progression of cancers (Leinonen et al., 2015; Lee et al., 2020). We also confirmed the high expression of NRF2 in our CRC tissues which might be caused by the overexpression of SLC7A11-AS1 in the present study.

In this study, we disclosed a positive correlation between the expression of SLC7A11-AS1 and SLC7A11 by bioinformatics analysis. We found that SLC7A11 and NRF2 were both increased in the CRC tissues with high SLC7A11-AS1 expression. Moreover, both of them significantly downregulated in HCT-8 cells when SLC7A11-AS1 was knocked down. However, the decreased SLC7A11 and ROS levels could be restored by NRF2 overexpression, which is caused by silencing SLC7A11-AS1. Therefore, SLC7A11-AS1 might upregulate its sense transcript, SLC7A11, expression by increasing the NRF2 protein level. As it has been proved that the lncRNA HIFAL recruited and directed the PKM2/PHDS complex to transactivate the expression of its sense transcript HIF-1 in breast cancer (Zheng et al., 2021). The promoter region of SLC7A11 could be directly bonded and activated by NRF2 to initiate transcription (Chen et al., 2017; Feng et al., 2021). The increased expression of SLC7A11 will lead to more genesis of glutathione, a primary antioxidant to clear ROS (Bader et al., 2021). However, SLC7A11-AS1 was reported to downregulate the expression of SLC7A11 in gastric cancer and epithelial ovarian cancer, in which the expression of SLC7A11-AS1 was decreased, and SLC7A11 was increased in the cancer tissues compared with the normal tissues (Yuan, Liu & Song, 2017; Luo et al., 2022). In our pan-cancer analysis based on the TCGA dataset, the expression of SLC7A11-AS1 was elevated in most kinds of cancers including stomach adenocarcinoma (STAD) and ovarian serous cystadenocarcinoma (OV). This discrepancy might be caused by the different patient cohorts, cancer types, and heterogeneity of tumors.

## Conclusions

In conclusion, we investigated the function of the lncRNA SLC7A11-AS1 in CRC for the first time. We found that silencing the expression of SLC7A11-AS1 would downregulate the NRF2/SLC7A11 signaling pathway resulting in increased ROS levels and suppressed proliferation, migration and invasion of CRC cells. Therefore, SLC7A11-AS1 might be a valuable diagnostic marker and therapeutic target for CRC.

##  Supplemental Information

10.7717/peerj.15216/supp-1 Supplemental Information 1 The wound healing assay were carried out to assess the effect of silencing SLC7A11-AS1 on HCT-8 cell migration

10.7717/peerj.15216/supp-2 Supplemental Information 2 The expression of NRF2 and SLC7A11 were downregulated by knockdown of SLC7A11-AS1 Total proteins of HCT-8 cells were collected after the treatment.

10.7717/peerj.15216/supp-3 Supplemental Information 3 The decreased expression of NRF2 in nucleus and SLC7A11 in cytoplasm after knocked down SLC7A11-AS1 expression

10.7717/peerj.15216/supp-4 Supplemental Information 4 NRF2 overexpression increased the expression of SLC7A11 after sliencing SLC7A11-AS1 expression

10.7717/peerj.15216/supp-5 Supplemental Information 5 NRF2 and SLC7A11 were upregulated in SLC7A11-AS1 overexpressed tissues

10.7717/peerj.15216/supp-6 Supplemental Information 6 Table S1. The result of qRT-PCR

10.7717/peerj.15216/supp-7 Supplemental Information 7 The comparison table of TCGA abbreviation of cancer names

10.7717/peerj.15216/supp-8 Supplemental Information 8 Quantity analysis of the proliferation, migration, invasion and ROS experiments

## Additional Information and Declarations

### Data Availability

The following information was supplied regarding data availability: The data is available at TCGA-COAD.
https://portal.gdc.cancer.gov/repository?facetTab=cases&filters=%7B%22op%22%3A%22and%22%2C%22content%22%3A%5B%7B%22op%22%3A%22in%22%2C%22content%22%3A%7B%22field%22%3A%22cases.primary_site%22%2C%22value%22%3A%5B%22colon%22%5D%7D%7D%2C%7B%22op%22%3A%22in%22%2C%22content%22%3A%7B%22field%22%3A%22cases.project.project_id%22%2C%22value%22%3A%5B%22TCGA-COAD%22%5D%7D%7D%2C%7B%22op%22%3A%22in%22%2C%22content%22%3A%7B%22field%22%3A%22files.access%22%2C%22value%22%3A%5B%22open%22%5D%7D%7D%2C%7B%22op%22%3A%22in%22%2C%22content%22%3A%7B%22field%22%3A%22files.data_category%22%2C%22value%22%3A%5B%22transcriptome%20profiling%22%5D%7D%7D%2C%7B%22op%22%3A%22in%22%2C%22content%22%3A%7B%22field%22%3A%22files.data_type%22%2C%22value%22%3A%5B%22Gene%20Expression%20Quantification%22%5D%7D%7D%2C%7B%22op%22%3A%22in%22%2C%22content%22%3A%7B%22field%22%3A%22files.experimental_strategy%22%2C%22value%22%3A%5B%22RNA-Seq%22%5D%7D%7D%5D%7D
