# Supplementary figures and images for: Downregulation of lncRNA SLC7A11-AS1 decreased the NRF2/SLC7A11 expression and inhibited the progression of colorectal cancer cells
Source: PeerJ. 2023 Apr 14;11:e15216. doi: 10.7717/peerj.15216 (PMC10108855; doi:10.7717/peerj.15216)

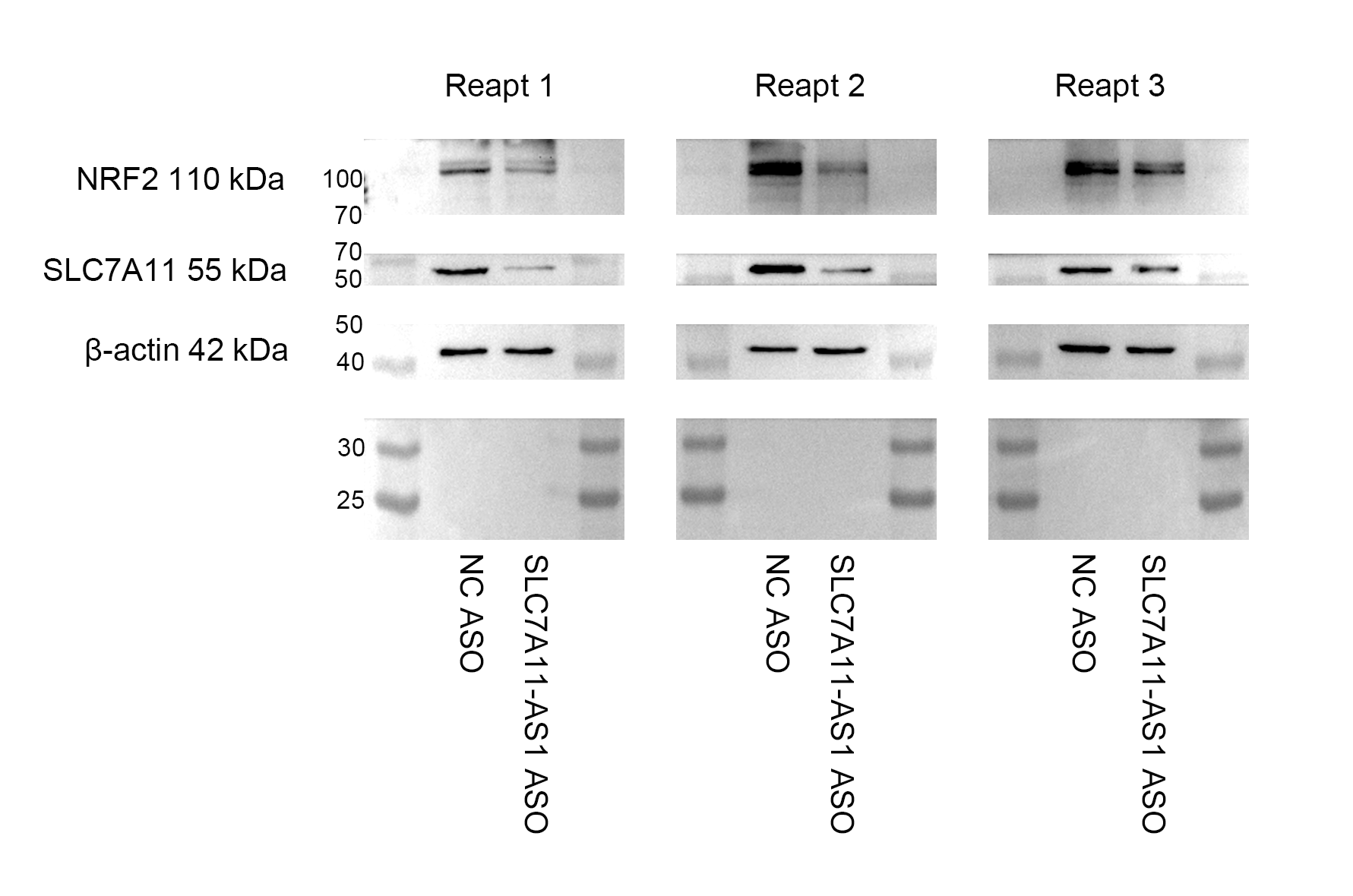

Supplement: Supplemental Information 1 [file peerj-11-15216-s001.png]

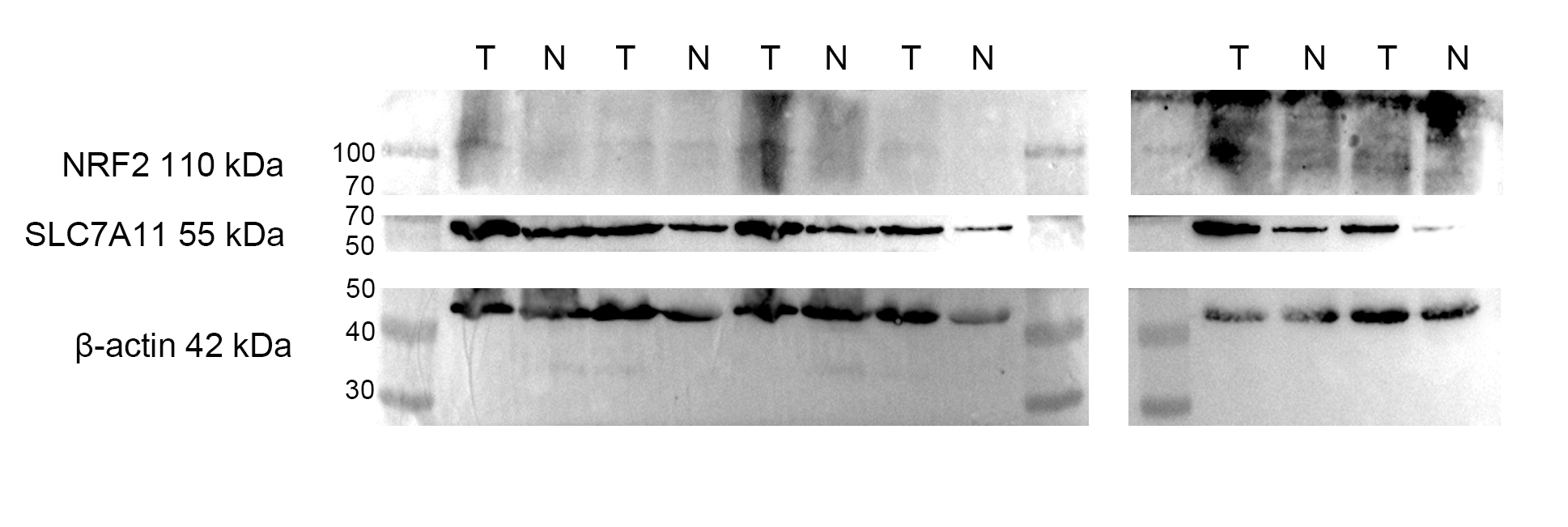

Supplement: Supplemental Information 2 — Total proteins of HCT-8 cells were collected after the treatment. [file peerj-11-15216-s002.png]

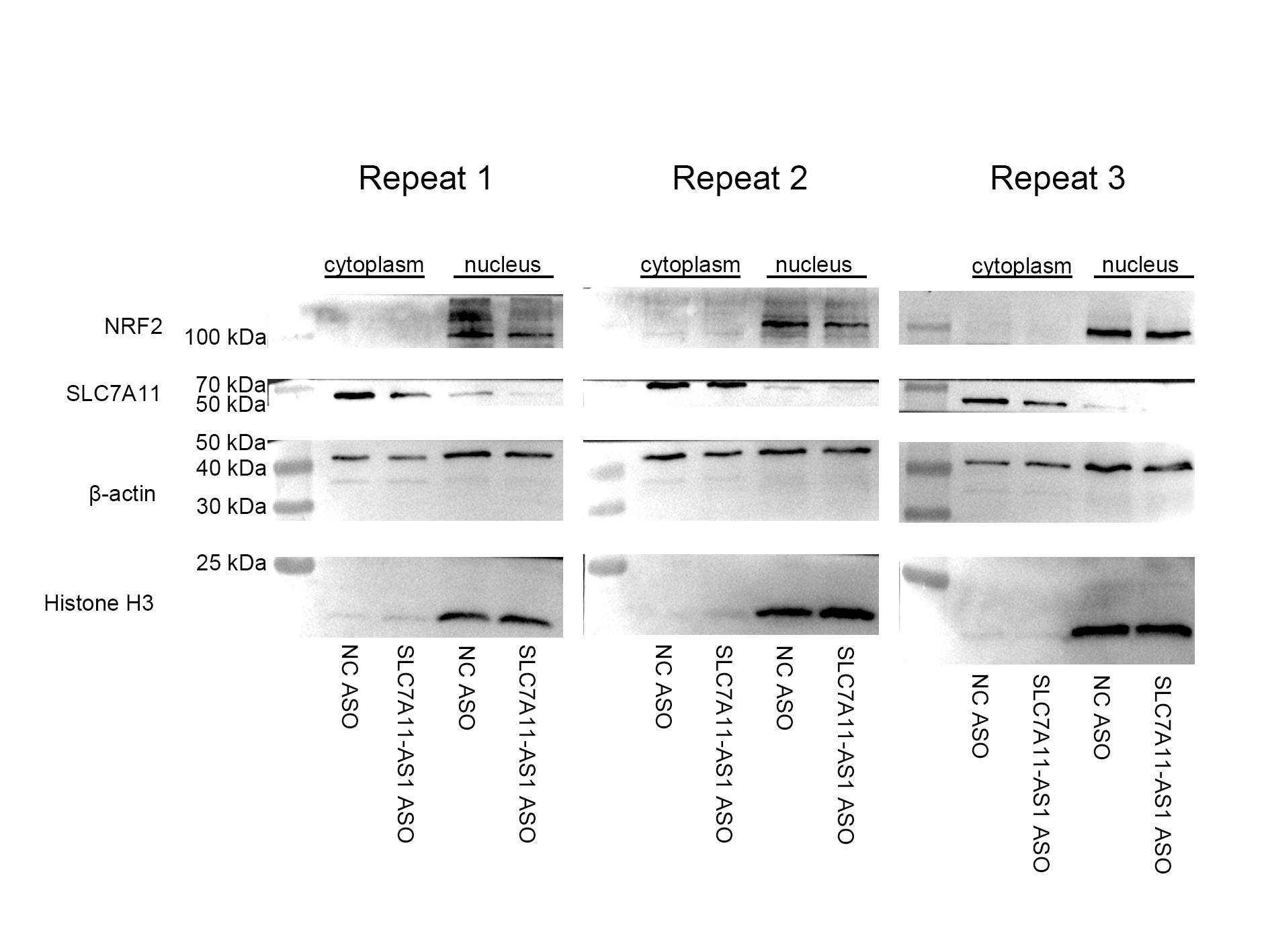

Supplement: Supplemental Information 3 [file peerj-11-15216-s003.png]

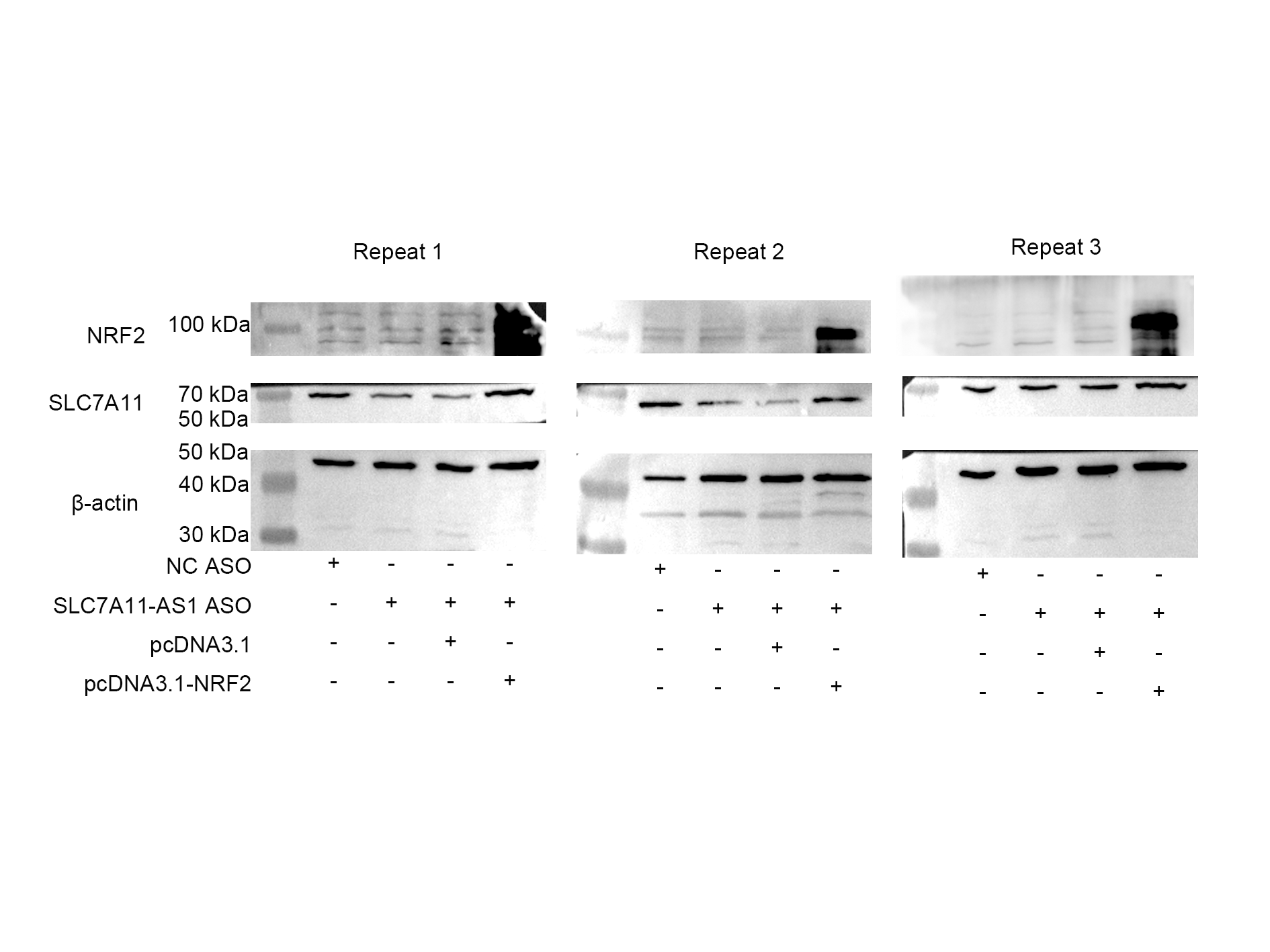

Supplement: Supplemental Information 4 [file peerj-11-15216-s004.png]

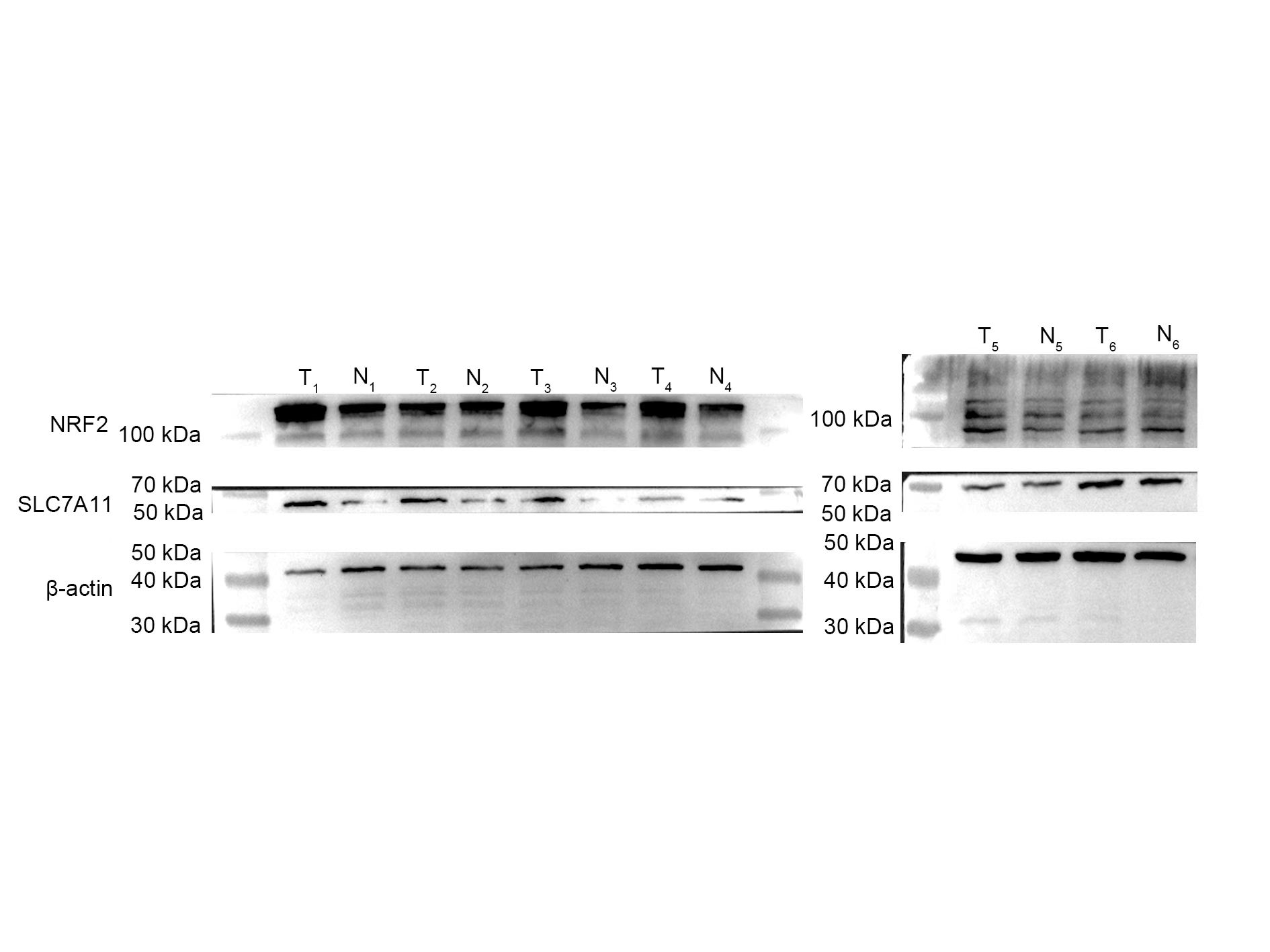

Supplement: Supplemental Information 5 [file peerj-11-15216-s005.png]
