# Supplementary material for: Downregulation of lncRNA SLC7A11-AS1 decreased the NRF2/SLC7A11 expression and inhibited the progression of colorectal cancer cells
Source: PeerJ. 2023 Apr 14;11:e15216. doi: 10.7717/peerj.15216 (PMC10108855; doi:10.7717/peerj.15216)
Supplement: Supplemental Information 7 [file peerj-11-15216-s007.docx]

| NO. | Abbreviation | Full name |
| --- | --- | --- |
| 1 | ACC | Adrenocortical   Carcinoma |
| 2 | BLCA | Bladder Urothelial   Carcinoma |
| 3 | BRCA | Breast Invasive   carcinoma |
| 4 | CESC | Cervical Squamous Cell Carcinoma and Endocervical Adenocarcinoma |
| 5 | CHOL | Cholangiocarcinoma |
| 6 | COAD | Colon Adenocarcinoma |
| 7 | DLBC | Lymphoid Neoplasm Diffuse Large B-cell Lymphoma |
| 8 | ESCA | Esophageal Carcinoma |
| 9 | GBM | Glioblastoma Multiforme |
| 10 | HNSC | Head and Neck Squamous Cell Carcinoma |
| 11 | KICH | Kidney Chromophobe |
| 12 | KIRC | Kidney Renal Clear Cell Carcinoma |
| 13 | KIRP | Kidney Renal Papillary Cell Carcinoma |
| 14 | LAML | Acute Myeloid Leukemia |
| 15 | LGG | Brain Lower Grade Glioma |
| 16 | LIHC | Liver Hepatocellular Carcinoma |
| 17 | LUAD | Lung Adenocarcinoma |
| 18 | LUSC | Lung Squamous Cell Carcinoma |
| 19 | MESO | Mesothelioma |
| 20 | OV | Ovarian Serous Cystadenocarcinoma |
| 21 | PAAD | Pancreatic Adenocarcinoma |
| 22 | PCPG | Pheochromocytoma and Paraganglioma |
| 23 | PRAD | Prostate Adenocarcinoma |
| 24 | READ | Rectum Adenocarcinoma |
| 25 | SARC | Sarcoma |
| 26 | SKCM | Skin Cutaneous Melanoma |
| 27 | STAD | Stomach Adenocarcinoma |
| 28 | TGCT | Testicular Germ Cell Tumors |
| 29 | THCA | Thyroid Carcinoma |
| 30 | THYM | Thymoma |
| 31 | UCEC | Uterine Corpus Endometrial Carcinoma |
| 32 | UCS | Uterine Carcinosarcoma |
| 33 | UVM | Uveal Melanoma |
